# Fast and Robust Monocular Visua-Inertial Odometry Using Points and Lines

**DOI:** 10.3390/s19204545

**Published:** 2019-10-19

**Authors:** Ning Zhang, Yongjia Zhao

**Affiliations:** State Key Laboratory of Virtual Reality Technology and Systems, School of Automation Science and Eletrical Engineering, Beihang University, Beijing 100191, China; zy1703239@buaa.edu.cn

**Keywords:** line feature, point-line feature fusion, semi-direct method

## Abstract

When the camera moves quickly and the image is blurred or the texture in the scene is missing, the Simultaneous Localization and Mapping (SLAM) algorithm based on point feature experiences difficulty tracking enough effective feature points, and the positioning accuracy and robustness are poor, and even may not work properly. For this problem, we propose a monocular visual odometry algorithm based on the point and line features and combining IMU measurement data. Based on this, an environmental-feature map with geometric information is constructed, and the IMU measurement data is incorporated to provide prior and scale information for the visual localization algorithm. Then, the initial pose estimation is obtained based on the motion estimation of the sparse image alignment, and the feature alignment is further performed to obtain the sub-pixel level feature correlation. Finally, more accurate poses and 3D landmarks are obtained by minimizing the re-projection errors of local map points and lines. The experimental results on EuRoC public datasets show that the proposed algorithm outperforms the Open Keyframe-based Visual-Inertial SLAM (OKVIS-mono) algorithm and Oriented FAST and Rotated BRIEF-SLAM (ORB-SLAM) algorithm, which demonstrates the accuracy and speed of the algorithm.

## 1. Introduction

Simultaneous Localization and Mapping (SLAM) [1,2,3] is used to incrementally estimate the pose of the mobile platform and simultaneously construct a map of the surrounding environment. Due to its ability to locate in an unknown environment, it is widely used in applications such as robot navigation [4,5,6] and Augmented Reality [7]. With the increasing demand for artificial intelligence and human-computer interaction, SLAM will play an increasingly important role in the future of science and technology. Robustness and real-time performance in challenging environments are two key factors in the application of this technology to engineering practice.

At present, the mainstream visual SLAM (vSLAM) scheme is divided into feature-based method [8,9,10], direct method [11,12,13], and semi-direct method [14] according to the way of using image information. Regarding the way of establishing the map, it is divided into sparse method [13], dense method [15], and semi-dense method [12]. The feature-based approach estimates the camera pose and constructs an environmental map by minimizing the reprojection errors observed and corresponding reprojection features. The most representative algorithms include Parallel Tracking And Mapping (PTAM) [9] and Oriented FAST and Rotated BRIEF-SLAM (ORB-SLAM) [10], which have achieved good performance in large scenes. The direct method does not need to extract salient features that can be repeatedly identified. Instead, it uses all the pixels with strong gradients in the image to optimize the pose by minimizing the photometric error to establish a dense or semi-dense environment map. Newcombe et al. proposed a completely straightforward method called Dense Tracking And Mapping (DTAM) [11]. DTAM tracks all the pixels of the entire image and builds a dense map of the environment that must be real-time on the GPU. Large-Scale Direct Monocular SLAM (LSD-SLAM) [12] is another dominant method in the direct approach. The core idea of LSD-SLAM follows the idea of semi-dense visual odometry. The semi-direct method only extracts features, does not calculate descriptors, does not perform feature matching, uses photometric error to establish data association between corresponding pixels, performs pose optimization, and uses the significant information in the image to establish sparse maps. Forster et al. proposed a more sparse semi-direct VO (SVO) [14]. It uses direct methods to track and triangulate pixels with high image gradients, but relies on a proven feature-based approach for joint optimization of structure and motion.

With the development of technology, more and more open-source systems have emerged, and visual SLAM technology has gradually matured. However, there are still many practical problems to be solved. Point features are widely used features in visual SLAM and are mature in feature extraction, matching, and representation. Common feature point description algorithms include Scale Invariant Feature Transform (SIFT) [16], Speeded Up Robust Features (SURF) [17], and Oriented FAST and Rotated BRIEF (ORB) [18]. However, point features are more dependent on the environment, and are not effective when the motion is too fast, resulting in blurred images and in weak textures scenes. In addition, the map constructed based on the point feature is a 3D point cloud map, and the point cloud map is sparse, but the map for robot navigation needs to reflect the structural information of the object in the scene, so as to study the path planning of the robot. Compared with point features, line features [19] are also widely present in various environmental scenarios, and line features are not susceptible to changes in viewpoints and illumination. Since the spatial dimension of a line is one-dimensional higher than a point, for some structured scenes, the line feature has an advantage and more accurately expresses the structural information of the environment [20,21]. The point feature [22,23] and the line feature [24,25,26] are complementary to each other. In weakly textured areas such as the ground and walls, almost no point features are extracted, but at the junction of the ground and the wall, there are abundant line segments. The environment map constructed by the combination of point and line features has more intuitive geometric information, and can also improve the accuracy and robustness of the SLAM system.

In addition, robot applications often require robots to be positioned in real-time [27]. When the robot moves quickly, the line feature extraction and tracking algorithm are relatively slow, which causes the movement and location to be out of synchronization. The longer the single location process, the less overlap between the two frames before and after, which will undoubtedly reduce the accuracy of the robot location. Compared with the feature-based method, the direct method does not need to detect and match features in each frame, and directly estimates the camera pose based on the photometric error of the corresponding points of adjacent frames, which greatly improves the running speed of the SLAM algorithm. Extending the line segment feature to the direct method can reduce the computational resource consumption and improve the robustness of the SLAM algorithm without significantly increasing the system computation time.

Although the indoor environment texture information is rich, it is inevitable that there will be no texture or weak texture. The existing solution is to combine visual and inertial [28] information for positioning, and according to the degree of fusion, it is loosely coupled [29,30] or tightly coupled [31,32,33]. The camera captures the rich information in the scene, and the IMU is able to obtain accurate estimates in a short time at high frequencies, mitigating the effects of dynamic objects on the camera [34]. In the absence of features, during fast motion, or in the case of dynamic obstacles, it is very helpful to use a priori from the IMU. A motion prior is an additional item attached to a cost function that penalizes motion that is inconsistent with a priori estimates. By using the complementarity of the inertial sensor and the image sensor, the pose estimation result with higher precision and better robustness can be obtained. In the literature [35], a novel tightly coupled monocular visual-inertial odometry algorithm PL-VIO is introduced, which optimizes the state of the system in a sliding window with point and line features. But this algorithm is a feature-based method, and has weak real-time performance.

In this paper, we present a visual-inertial odometry that combines point and line features. This algorithm is based on an extension of the semi-direct method SVO. First, line features provide more geometric information about the environment than point features. Line features help improve system robustness in challenging scenarios, such as low-texture environments or lighting variations, when point features cannot be reliably detected or tracked. Based on the extension of the direct method, the extraction and matching of the line segment features are reduced, and a faster speed can be achieved. The second extension, combined with motion prior information, allows the system to be robust in environments with the lack of features or fast motion, while at the same time restoring the precise scale of the camera pose.

## 2. Methodology

**Figure 1:**
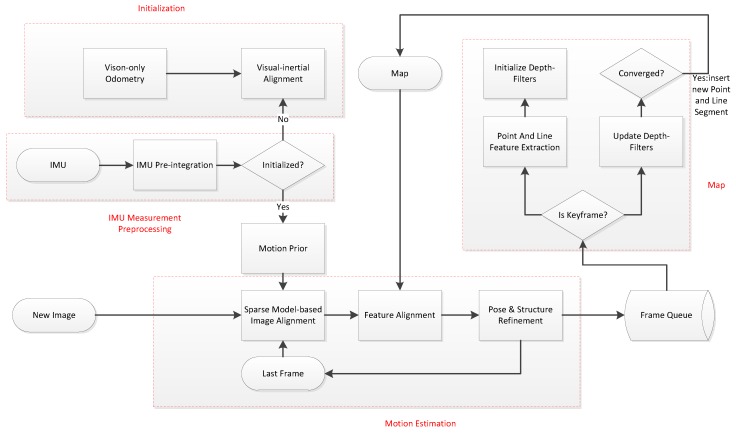
Flow chart of the proposed algorithm.

Figure 1 shows the overall framework of our approach, adding line segment features and motion prior extensions to SVO. The whole algorithm includes four modules: IMU measurement preprocessing, visual-inertial initialization, motion estimation, and mapping.

The measurement pre-processing module pre-integrates the IMU measurement data between consecutive frames. In the visual inertia alignment module, the SFM is first used to estimate the pose and 3D point inverse depth of all frames in the sliding window, and then the IMU pre-integration is used to solve the initialization parameters. The motion estimation module performs point and line segment feature tracking based on the direct method, and combines the motion prior information to estimate the camera pose. Finally, the mapping thread recursively estimates the 3D position of the image features with an unknown depth.

### 2.1. Notations

We define some of the notations and coordinate system definitions needed in this paper. ${\left(\cdot \right)}^{w}$ represents the world coordinate system. ${\left(\cdot \right)}^{b}$ represents the IMU and body coordinate system. ${\left(\cdot \right)}^{c}$ represents the camera coordinate system. We use rotation matrices R and quaternions q to represent rotation, and p represents translation. $q_{b}^{w}$ and $p_{b}^{w}$ are rotation and translation from the body frame to the world frame. $b_{k}$ is the body frame while taking the *k*th image. $c_{k}$ is the camera frame while taking the *k*th image. ⊗ represents the multiplication operation.

The image acquired from one camera *C* at time *k* is denoted as $I_{k}^{c}:\Omega^{c}\subset R^{2}$, where $\Omega^{c}$ is the image field. Any 3D point $\rho \in R^{3}$ projection to image coordinates $u\in R^{2}$ through the camera projection model: $u=\pi \left(\rho \right)$. Given the inverse scene depth d>0 at pixel $u\in R_{k}^{c}$, the position of the 3D point is obtained using the inverse projection model $\rho =\pi^{-1}\left(u,d\right)$. Among them, we use $R_{k}^{c}\subseteq \Omega^{c}$ to represent the pixel point whose depth is known in camera *C* at time *k*.

In the case of a line segment, we use the two endpoints of the line segment to represent a line segment feature l with p and q, respectively. In order to introduce a motion prior constraint, we define a body coordinate system b that is rigidly connected to the camera frame c, and this external calibration parameters $T_{CB}\in SE\left(3\right)$ are known in the provided datasets or calibrated with the Kalibr calibration toolbox [36]. The line segment feature whose endpoint depth is known in camera C at time *K* is represented by $L_{k}^{c}\subseteq \Omega^{c}$, and the endpoints of the line segment feature are denoted by p and q. The projection model is obtained by pre-calibrating the camera.

The position and rotation of the world frame W relative to the kth camera frame can be described by the rigid body transformation $T_{kw}\in SE\left(3\right)$. A 3D point ${}_{w}\rho$ expressed in the world coordinate system can be transformed to the kth camera: ${}_{k}\rho =T_{kw}\cdot {}_{w}\rho$.

### 2.2. Visual-Inertial Initialization

The main purpose of the visual-inertial odometry system initialization [37,38] is to obtain the parameters necessary for the system to optimize and the initial value of the state variables. Since the monocular inertial odometry system is a system with a high degree of nonlinearity, the quality of the initialization directly affects the robustness of the entire tightly coupled system and the location accuracy. Therefore, it is necessary to initialize the system in a specific way to provide accurate parameters and initial values.

In the process of initialization, the information that needs to be initialized or estimated can be divided into two categories: (1) Parameters that are almost constant or have little change during system operation, such as absolute scale and gravitational acceleration; (2) The initial value of the system starting state quantity, including the pose and velocity information of the first few keyframes and the position of the 3D landmark points, and the bias of the IMU accelerometer and the gyroscope.

**Figure 2:**
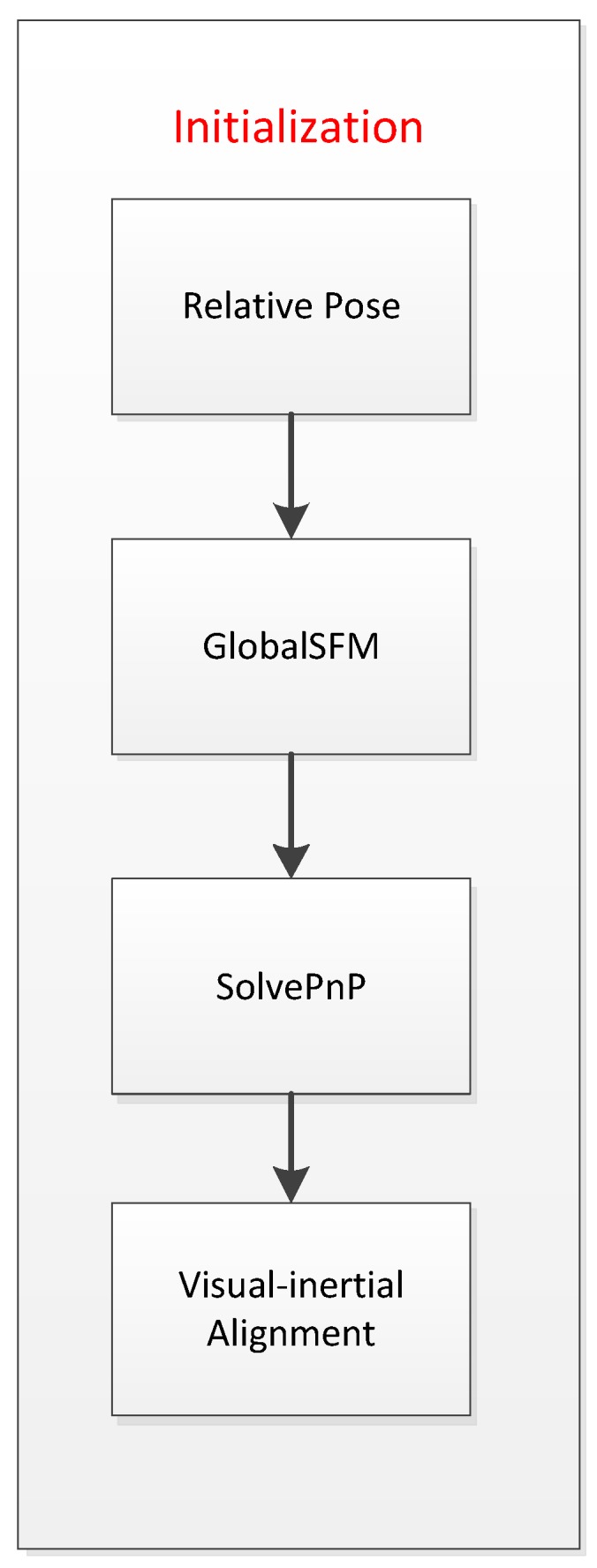
Initialization flow chart.

We use the initialization method proposed in [38]. The initialization method of the algorithm in this paper is divided into two processes. First, the initial visual keyframes are tracked using the semi-direct method of pure vision. The semi-direct method monocular visual odometry can initialize the initial keyframes pose information and the 3D landmark point position information changed with scale. The second process is visual-inertial alignment, which can initialize precise scales, gravitational acceleration, speed information of the camera state, and zero offsets of the accelerometer and gyroscope. The processing of the IMU data is to calculate the pre-integration result between adjacent keyframes, input to the visual-inertial alignment module for an initial solution, solve an accurate scale, and convert the results of the pose estimation into the IMU coordinate system. The initialization flow chart is shown in Figure 2.

As shown in Figure 2, a keyframe with a large number of matching feature points of the current frame is searched in the sliding window as a reference frame, and the relative pose of the current frame to the reference frame is calculated by solving the fundamental matrix. Next, SFM is performed on these two keyframes to obtain the camera pose and feature position of any scale. For each frame of the image in the sliding window, the solvePnP [39] is performed to get pose with all the 3D landmark points obtained by the SFM.

At this point, the pose information of all keyframes and the 3D information of the points and line segments can be obtained. Since the external parameters $\left(q_{c}^{b},p_{c}^{b}\right)$ between the camera and the IMU are known, all variables can be transformed into the IMU coordinate system to represent:

(1) $$\begin{array}{cc} q_{b_{k}}^{w} & =q_{c_{k}}^{w}⊗{\left(q_{c}^{b}\right)}^{-1}\\ sp_{b_{k}}^{w} & =sp_{c_{k}}^{w}-R_{b_{k}}^{w}p_{c}^{b} \end{array},$$

where *s* is an unknown scale factor. According to the method in [38], the vision-only pose estimation result and the IMU measurement pre-integration are visual-inertial aligned, and the absolute scale, the gravity acceleration, the speed information of the camera state, and the zero offsets of the IMU can be estimated.

Since the IMU pose estimation data is of absolute scale, the camera pose estimation is not drifting. After the two are aligned, the absolute scale of the camera pose can be well estimated. At this point, the initialization process is complete.

### 2.3. IMU Measurement Pre-Integration

**Figure 3:**
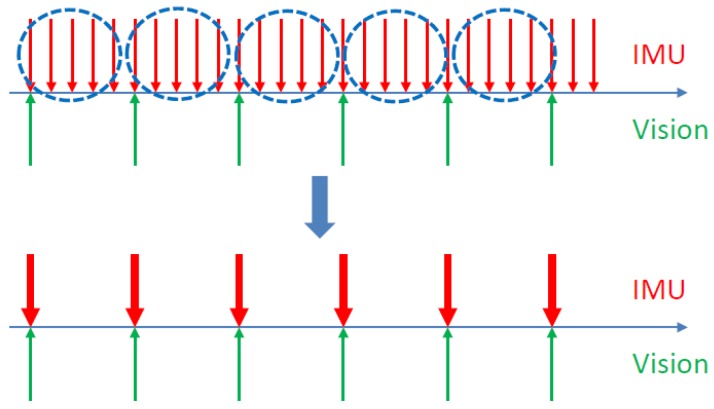
IMU Pre-integration diagram.

The IMU consists of a three-axis accelerometer and a three-axis gyroscope that measure angular velocity and acceleration relative to the inertial coordinate system. Since the measurement frequency of the IMU is much faster than that of the vision camera, as shown in Figure 3, it is desirable to incorporate constraints from inertial measurements into the motion estimation, which requires integrating the measurements of the numerous IMU data of two adjacent visual keyframes into one constraint. The manifold-based pre-integration theory adopted in this paper was proposed by Forster et al. in 2016 [40], which uses the IMU pre-integration method to transform IMU measurement data into visual keyframe constraints.

The state variables at time *k* of the IMU coordinate system *B* are defined as the position $p_{b_{k}}^{w}$, velocity $v_{b_{k}}^{w}$, and rotation $q_{b_{k}}^{w}$. All accelerometer and gyroscope measurement data between time *k* and time k+1 are given. At the time k+1, the position $p_{b_{k+1}}^{w}$, velocity $v_{b_{k+1}}^{w}$ and rotation $q_{b_{k+1}}^{w}$ are calculated by integrating all the IMU data between time *k* and time k+1, and are defined as the initial values of the visual estimation.

(2) $$\begin{array}{cc} p_{b_{k+1}}^{w} & =p_{b_{k}}^{w}+v_{b_{k}}^{w}\Delta t_{k}+\underset{t\in \left[k,k+1\right]}{\;\int \int \;}\left[R_{t}^{w}\left({\hat{a}}_{t}-b_{a_{t}}\right)-g^{w}\right]dt^{2}\\ v_{b_{k+1}}^{w} & =v_{b_{k}}^{w}+\int_{t\in \left[k,k+1\right]}\left[R_{t}^{w}\left({\hat{a}}_{t}-b_{a_{t}}\right)-g^{w}\right]dt\\ q_{b_{k+1}}^{w} & =q_{b_{k}}^{w}⊗\int_{t\in \left[k,k+1\right]}\frac{1}{2}\Omega \left({\hat{w}}_{t}-b_{w_{t}}\right)q_{t}^{b_{k}}dt \end{array},$$

where ${\hat{a}}_{t}$ and ${\hat{w}}_{t}$ are the acceleration and angular velocity measured by the IMU. $g^{w}$ is the gravity vector in the world frame. $b_{a_{t}}$ is the acceleration bias and $b_{w_{t}}$ is the gyroscope bias. In practical use, the IMU data is discrete, and the discrete formula based on the median integration method is given below. That is the integration process from the time *i* to the time i+1.

(3) $$\begin{array}{cc} p_{b_{i+1}}^{w} & =p_{b_{i}}^{w}+v_{b_{i}}^{w}\delta t+\frac{1}{2}{\bar{\hat{a}}}_{i}\delta t^{2}\\ v_{b_{i+1}}^{w} & =v_{b_{i}}^{w}+{\bar{\hat{a}}}_{i}\delta t\\ q_{b_{i+1}}^{w} & =q_{b_{i}}^{w}⊗\left[\begin{array}{c} 1\\ \frac{1}{2}{\bar{\hat{w}}}_{i}\delta t \end{array}\right] \end{array},$$

where:

(4) $$\begin{array}{cc} {\bar{\hat{a}}}_{i} & =\frac{1}{2}\left[q_{i}\left({\hat{a}}_{i}-b_{a_{i}}\right)-g^{w}+q_{i+1}\left({\hat{a}}_{i+1}-b_{a_{i}}\right)-g^{w}\right]\\ {\bar{\hat{w}}}_{i} & =\frac{1}{2}\left({\hat{w}}_{i}+{\hat{w}}_{i+1}\right)-b_{w_{i}} \end{array}.$$

By observing the Formula (2), the pre-integration of the IMU needs to depend on the v and R of the *k*th frame. When we perform nonlinear optimization on the backend, we need to iteratively update the v and R of the *k*th frame. We need to re-integrate based on the value after each iteration, which is very time-consuming. Therefore, we consider separating the optimization variable from the IMU pre-integration term from the *k* frame to the k+1 frame. By multiplying the left and right sides of the Formula (2) by $R_{w}^{b_{k}}$, it can be reduced to:

(5) $$\begin{array}{cc} R_{w}^{b_{k}}p_{b_{k+1}}^{w} & =R_{w}^{b_{k}}\left(p_{b_{k}}^{w}+v_{b_{k}}^{w}\Delta t_{k}-\frac{1}{2}g^{w}\Delta t_{k}^{2}\right)+\alpha_{b_{k+1}}^{b_{k}}\\ R_{w}^{b_{k}}v_{b_{k+1}}^{w} & =R_{w}^{b_{k}}\left(v_{b_{k}}^{w}-g^{w}\Delta t_{k}\right)+\beta_{b_{k+1}}^{b_{k}}\\ q_{w}^{b_{k}}⊗q_{b_{k+1}}^{w} & =\gamma_{b_{k+1}}^{b_{k}} \end{array},$$

where:

(6) $$\begin{array}{cc} \alpha_{b_{k+1}}^{b_{k}} & ={\;\int \int \;}_{t\in \left[k,k+1\right]}\left[R_{t}^{b_{k}}\left({\hat{a}}_{t}-b_{a_{t}}\right)\right]dt^{2}\\ \beta_{b_{k+1}}^{b_{k}} & =\int_{t\in \left[k,k+1\right]}\left[R_{t}^{b_{k}}\left({\hat{a}}_{t}-b_{a_{t}}\right)\right]dt\\ \gamma_{b_{k+1}}^{b_{k}} & =\int_{t\in \left[k,k+1\right]}\frac{1}{2}\Omega \left({\hat{w}}_{t}-b_{w_{t}}\right)\gamma_{t}^{b_{k}}dt \end{array}.$$

In this way, we obtain the IMU pre-integration formula for continuous-time. It can be found that the value of the IMU pre-integration obtained by the above formula is only related to ${\hat{a}}_{t}$ and ${\hat{w}}_{t}$ at different times.

Similarly, we give the IMU pre-integration formula for discrete moments based on the median integration method. The IMU increment information from time *i* to time i+1 is:

(7) $$\begin{array}{cc} {\hat{\alpha }}_{i+1}^{b_{k}} & ={\hat{\alpha }}_{i}^{b_{k}}+{\hat{\beta }}_{i}^{b_{k}}\delta t+\frac{1}{2}{\bar{\hat{a}}}_{i}\delta t^{2}\\ {\hat{\beta }}_{i+1}^{b_{k}} & ={\hat{\beta }}_{i}^{b_{k}}+{\bar{\hat{a}}}_{i}\delta t\\ {\hat{\gamma }}_{i+1}^{b_{k}} & ={\hat{\gamma }}_{i}^{b_{k}}⊗{\hat{\gamma }}_{i+1}^{i}={\hat{\gamma }}_{i}^{b_{k}}⊗\left[\begin{array}{c} 1\\ \frac{1}{2}{\bar{\hat{w}}}_{i}\delta t \end{array}\right] \end{array},$$

where:

(8) $$\begin{array}{cc} {\bar{\hat{a}}}_{i} & =\frac{1}{2}\left[q_{i}\left({\hat{a}}_{i}-b_{a_{i}}\right)-g^{w}+q_{i+1}\left({\hat{a}}_{i+1}-b_{a_{i}}\right)-g^{w}\right]\\ {\bar{\hat{w}}}_{i} & =\frac{1}{2}\left({\hat{w}}_{i}+{\hat{w}}_{i+1}\right)-b_{w_{i}} \end{array}.$$

## 3. Visual Odometry Combined with Point and Line Features

The main purpose of this study is to develop a more robust semi-direct method SLAM algorithm combining point and line features. The algorithm achieves the same accuracy as the most advanced feature-based methods and maximizes the speed of the algorithm so that it can be used for a variety of lightweight platform tasks such as cell phones and micro drones.

### 3.1. Monocular Initialization

We use a feature-based approach to obtain the pose and initial map of the initial two keyframes. First, the Fast corner feature and the LSD segment feature are extracted on the first keyframe, and then the features on the first keyframe are tracked using the klt optical flow algorithm [41]. The disparity between the two frames is calculated until the image that reaches the set disparity threshold is selected as the second keyframe. We calculate the homography matrix by the local plane hypothesis to obtain the pose transformation of the second keyframe relative to the first keyframe, and obtain reliable inlier matching. An initial map of random scales is obtained by triangulation between the initial two keyframes. With the pose and map of the initial two keyframes, the direct method can be used to estimate the pose of the new frame.

### 3.2. Sparse Model-Based Image Alignment with Motion Prior

The motion between two consecutive camera frames can be estimated by direct tracking of sparse features. By minimizing the photometric error of the corresponding pixels between two consecutive camera frames, we can get an initial estimate of the pose between two adjacent frames. We need to define the photometric cost function of the point and line segment features separately. Model-based image alignment estimates the pose increment between adjacent frames by minimizing the intensity difference (photometric error) of pixels viewing the same 3D point and line segment.

Our goal is to estimate the incremental motion of the body coordinate system $T_{kk-1}\overset{.}{\;=}T_{B_{k}B_{k-1}}$. Next, we define the residual functions corresponding to points, line segments, and motion priors. Define the intensity residuals of the point features $\delta I_{p}$ as:

(9) $$\delta I_{p}\left(T_{kk-1},u\right)=r_{I_{u_{i}}^{C}}\left(T_{kk-1}\right),$$

where the photometric residual is defined by the intensity difference of the pixels of the same 3D point $\rho_{i}$ observed in subsequent images $I_{k}^{c}$ and $I_{k-1}^{c}$.

(10) $$r_{I_{u_{i}}^{c}}\left(T_{kk-1}\right)=I_{k}^{c}\left(\pi \left(T_{CB}T_{kk-1}\rho_{i}\right)\right)-I_{k-1}^{c}\left(\pi \left(T_{CB}\rho_{i}\right)\right).$$

The 3D point $\rho_{i}$ (represented in the reference frame $B_{k-1}$) can be calculated by back-projection pixels with a known depth $d_{i}$:

(11) $$\rho_{i}=T_{BC}\pi^{-1}\left(u_{i},d_{i}\right),\forall u_{i}\in R_{k-1}^{c}.$$

But the optimization in Equation (9) only includes a subset of these pixels ${\bar{R}}_{k-1}^{c}\subseteq R_{k-1}^{c}$, indicating that these back-projection points are also visible in the image $I_{k}^{c}$:

(12) $${\bar{R}}_{k-1}^{c}=\left\{u\left|u\in R_{k-1}^{c}\wedge \pi \left(T_{CB}T_{kk-1}T_{BC}\pi^{-1}\left(u,d_{u}\right)\right)\in \Omega_{k}^{c}\right.\right\}.$$

**Figure 4:**
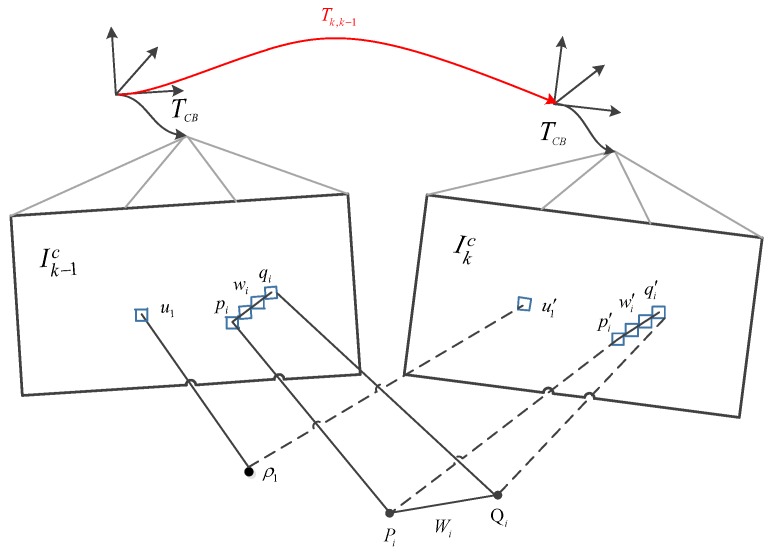
Relative pose between the current frame and the previous frame parameterizes the position of the reprojection point in the new image. Perform sparse image alignment to minimize luminosity error between image blocks corresponding to the same 3D point (blue block) to solve pose increment $T_{kk-1}$.

Unlike the point-based approach, we cannot directly align the entire area occupied by a line segment between two frames because it is computationally expensive. To this end, we only minimize the image residuals between patches that are evenly distributed along the line segment, as shown in Figure 4. We define ${\bar{L}}_{k-1}^{c}$ as the image region where the endpoint depth is known at the previous time k−1, and at the current time *k*, the endpoints p and q are visible in the image domain $\Omega_{k}^{c}$.

(13) $$\begin{array}{c} {\bar{L}}_{k-1}^{c}:=\left\{p,q,w_{n}\left|p,q\in L_{k-1}^{c}\wedge \pi \left(T_{CB}T_{kk-1}T_{BC}\cdot \pi^{-1}\left(p,d_{p}\right)\right)\in \Omega_{k}^{c}\right.\right.\\ \left.\wedge \pi \left(T_{CB}T_{kk-1}T_{BC}\cdot \pi^{-1}\left(q,d_{q}\right)\right)\in \Omega_{k}^{c}\right\} \end{array},$$

where $w_{n},n=2,\ldots ,N_{l}-1$ refers to the intermediate point defined evenly along the line segment.

The intensity residual of line segment $\delta I_{l}$ is then defined as the photometric difference between the pixels of the same 3D line segment point, i.e.,:

(14) $$\delta I_{l}\left(T_{kk-1},l\right)=\frac{1}{N_{l}}\sum_{n=0}^{N_{l}}\left|I_{k}^{c}\left(\pi \left(T_{CB}T_{kk-1}w_{n}\right)\right)-I_{k-1}^{c}\left(T_{CB}w_{n}\right)\right|.$$

In the case of n=0 and $n=N_{l}$, the point $w_{n}$ refers to the endpoints *p* and *q* respectively.

We further assume that the a prior ${\tilde{T}}_{kk-1}\dot{=}\left(\tilde{R},\tilde{p}\right)$ of the body coordinate system motion increment is given. In this case, we define the residuals corresponding to the rotation prior and the translation prior:

(15) $$\begin{array}{cc} r_{R} & =\log{\left({\tilde{R}}_{kk-1}^{T}R_{kk-1}\right)}^{\vee }\\ r_{p} & =p_{kk-1}-{\tilde{p}}_{kk-1} \end{array}.$$

In order to jointly calculate the optimal pose increment, we unify the point features, line segment features, and motion prior residuals into a least-squares optimization cost function. The goal is to solve for incremental camera rotation and translation $T_{kk-1}=\left(R,p\right)$ by minimizing the sum of the squared errors below:

(16) $$\left(R^{*},p^{*}\right)=\arg\min_{\left(R,p\right)}r\left(R,p\right),$$

where:

(17) $$r\left(R,p\right)=\sum_{u_{i}\in {\bar{R}}_{k-1}^{c}}\frac{1}{2}{∥\delta I_{p}\left(T_{kk-1},u_{i}\right)∥}_{\sum_{I}}^{2}+\sum_{l_{j}\in {\bar{L}}_{k-1}^{c}}\frac{1}{2}{∥\delta I_{l}\left(T_{kk-1},l_{j}\right)∥}_{\sum_{I}}^{2}+\frac{1}{2}{∥r_{R}∥}_{\Sigma_{R}}^{2}+\frac{1}{2}{∥r_{p}∥}_{\sum_{p}}^{2},$$

where the covariance $\sum_{p}$, $\sum_{R}$ is set according to the uncertainty of the motion prior, and the variable $\left(R_{kk-1},p_{kk-1}\right)$ is the current estimate of the relative position and rotation (represented in the B frame). A logarithmic map transforms a rotation matrix to its rotation vector.

To facilitate the solution, we write the cost function in matrix form.

(18) $$C\left(R,p\right)=r{\left(R,p\right)}^{T}\Sigma^{-1}r\left(R,p\right),$$

where Σ is a block diagonal matrix consisting of measurement covariances. The solution to the optimization variable is non-linear, equivalent to solving a least-squares problem. We use the iterative method Gauss-Newton to solve this problem, adding the following perturbations to the optimization variable rotation R and translation p:

(19) $$R^{\prime }=R*\exp\left(\delta \phi^{\wedge }\right),p^{\prime }=p+R\delta p.$$

The operator ${\left(.\right)}^{\wedge }$ turns a three-dimensional vector into an orthogonal matrix of 3 × 3, where ϕ is a Lie algebra. The perturbation form is used to define the residual function in the vecto space. This allows us to linearize the current estimated quadratic costs, form normal equations, and solve them for the best perturbations:

(20) $$J^{T}\Sigma^{-1}J{\left[\delta \phi^{T}\delta p^{T}\right]}^{T}=-J^{T}\Sigma^{-1}r\left(R,p\right).$$

We introduce the matrix J, which superimposes all Jacobian matrices from linearization. The solution result is then used to update the estimate of $T_{kk-1}=\left(R,p\right)$ according to Equation (19). This process is repeated until the norm of the update vector is small enough, which indicates convergence.

In the following, we give the Jacobian matrix obtained by linearizing the residual:

(21) $$\begin{array}{cc} \frac{\partial r_{R}}{\partial \delta \phi } & =J_{r}^{-1}\left(\log\left({\tilde{R}}^{T}R\right)\right)\\ \frac{\partial r_{p}}{\partial \delta p} & =R\\ \frac{\partial r_{I_{u_{i}}^{C}}}{\partial \delta \phi } & =-\frac{\partial I_{k-1}^{C}\left(u\right)}{\partial u}\left|{}_{{}_{u=\pi \left(c\rho_{i}\right)}}\right.\frac{\partial \pi \left(\rho \right)}{\partial \rho }\left|{}_{\rho =c\rho_{i}}\right.R_{CB}\rho_{i}^{\wedge }\\ \frac{\partial r_{I_{{}_{u_{i}}}^{C}}}{\partial \delta p} & =-\frac{\partial I_{k-1}^{C}\left(u\right)}{\partial u}\left|{}_{{}_{u=\pi \left(c\rho_{i}\right)}}\right.\frac{\partial \pi \left(\rho \right)}{\partial \rho }\left|{}_{\rho =c\rho_{i}}\right.R_{CB} \end{array},$$

where $J_{r}^{-1}$ is the inverse of the SO(3) right Jacobian matrix, $\frac{\partial I_{k-1}^{C}\left(u\right)}{\partial u}$ is the image derivative at pixel **u**, and $\frac{\partial \pi \left(\rho \right)}{\partial \rho }$ is the derivative of the camera projection model. For the standard pinhole camera projection model focal length $\left(f_{x},f_{y}\right)$ and camera center $\left(c_{x},c_{y}\right)$, we define:

(22) $$\frac{\partial \rho \left(\rho \right)}{\partial \rho }=\left[\begin{array}{ccc} \frac{f_{x}}{z} & 0 & -f_{x}\frac{x}{z^{2}}\\ 0 & \frac{f_{y}}{z} & -f_{y}\frac{y}{z^{2}} \end{array}\right],\rho ={\left[x,y,z\right]}^{T}.$$

(23) $$J_{r}^{-1}\left(\phi \right)=I+\frac{1}{2}\phi^{\wedge }+\left(\frac{1}{{∥\phi ∥}^{2}}+\frac{1+\cos\left(∥\phi ∥\right)}{2∥\phi ∥\sin\left(∥\phi ∥\right)}\right){\left(\phi^{\wedge }\right)}^{2}.$$

In this case, we look for the linear Jacobian determinant of the line segment residual, which can be expressed as the sum of the Jacobian determinants for each intermediate point $w_{n}$ of the sample:

(24) $$\begin{array}{cc} \frac{\partial r_{I_{l_{i}}^{C}}}{\partial \delta \phi } & =\frac{1}{N_{l}}\sum_{n=0}^{N_{l}}\frac{\partial r_{I_{w_{n}}^{C}}}{\partial \delta \phi }\\ \frac{\partial r_{I_{{}_{l_{i}}}^{C}}}{\partial \delta p} & =\frac{1}{N_{l}}\sum_{n=0}^{N_{l}}\frac{\partial r_{I_{w_{n}}^{C}}}{\partial \delta p} \end{array}.$$

Then, we can estimate the optimal pose by the robust Gaussian Newton minimization of the above cost function in Equation (18). Note that this formula allows for fast-tracking of line segments without the need to extract and match LSD segment features, and the traditional feature-based approach introduces high computational load.

### 3.3. Feature Alignment

In the previous step, we estimated the incremental motion between successive frames by sparse image alignment. With the known pose $T_{kk-1}$, we can reproject all visible 3D features into the new image to get an initial estimate of the position. Because of the inaccuracy of the 3D feature position, the feature position in the new image can be improved. In order to reduce the drift of the pose, the camera pose should be further aligned with the map, not just the previous frame. The feature alignment method selects the older keyframes as a reference for feature alignment, ignoring the geometric constraints given by the re-projection of the 3D points and performing a separate 2D alignment of the corresponding feature blocks.

**Figure 5:**
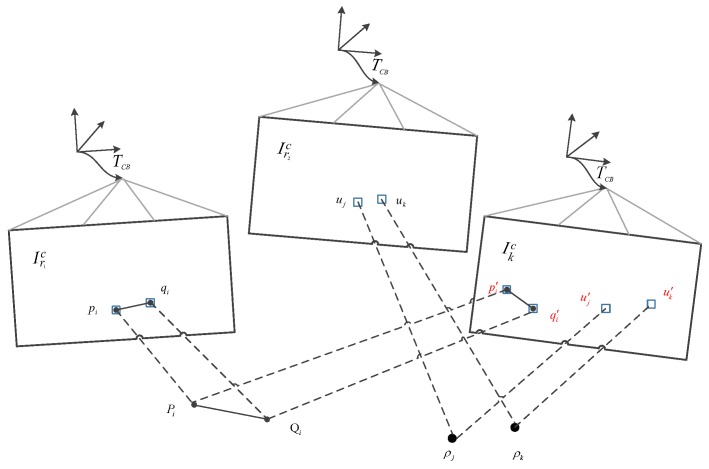
The 2D position of each point is individually optimized to minimize photometric errors in its image block. For line segments, the endpoints are similarly optimized.

All 3D points in the map visible in the new image are projected onto the image plane, resulting in a corresponding 2D feature position estimate $u_{i}^{\prime }$ (as shown in Figure 5). For each re-projection feature, the visual keyframe closest to the new image is determined to be the reference frame. Next, all 2D feature positions are respectively optimized by establishing photometric errors of the feature blocks in the new image and the feature blocks in the corresponding keyframe *r*. The 2D feature alignment minimizes the intensity difference of the small image block P centered on the feature projection position u. The size of the P of the image block is a larger 8 pixels × 8 pixels because the closest keyframe we project the feature is usually farther away. In order to improve the accuracy of the alignment, we apply the affine warping A to the reference block, which is calculated from the relative estimated pose $T_{kr}$ between the reference frame and the current frame. For the corner feature, the optimization calculates the correction of the predicted feature position $u^{\prime }$, minimizing the photometric cost function:

(25) u′*=u′+δu*,with u′=πTCBTkrTBCπd−1u.

(26) $$\delta u^{*}=\underset{\delta u}{\arg\min}\sum_{\Delta u\in P}\frac{1}{2}{∥I_{k}^{c}\left(u^{\prime }+\delta u+\Delta u\right)-I_{r}^{c}\left(u+A\Delta u\right)∥}^{2},$$

where Δu is an iterator variable that is used to calculate the sum of patch P. This alignment is solved using the inverse component Lucas-Kanade algorithm.

In the case of a line segment, we only need to refine the position of the 2D endpoint, which defines the line equation used for projection error estimation:

(27) $$w_{j}^{\prime }=\underset{{w^{\prime }}_{j}}{\arg\min}{∥I_{k}^{c}\left({w^{\prime }}_{j}\right)-I_{r}^{c}\left(A_{j}\cdot w_{j}\right)∥}^{2},\forall j,$$

where $w_{j}^{\prime }$ is the two-dimensional estimated position of the feature in the current frame ($p_{j}^{\prime },q_{j}^{\prime }$ representing the two endpoints of the line segment, respectively), and $w_{j}$ is the position of the feature in the reference frame *r*. For line segments, this is a bold assumption because their endpoints are much less than the description of the key points.

In comparison with the feature-based approach, in this step we skip the limits of the polar constraint, but achieve the feature correlation of subpixel precision.

### 3.4. Pose and Structure Refinement

**Figure 6:**
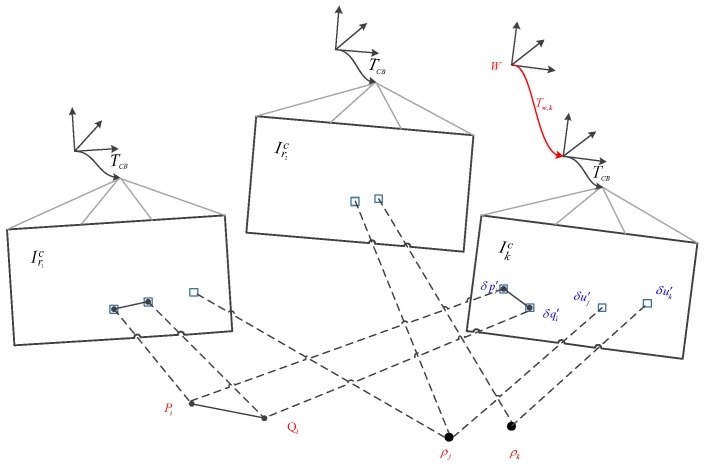
In the final step of motion estimation, the camera pose and structure (3D points and line segments) are optimized to minimize the reprojection errors established in the previous feature alignment steps.

After feature alignment, we established feature correlations related to subpixel precision. However, feature alignment violates the epipolar line constraint and introduces a reprojection error δu. After separately optimizing the position of each feature in the image by skipping the epipolar line constraint, the camera pose obtained in Formula (16) must be further refined by minimizing the reprojection error between the 3D feature and the corresponding 2D feature position in the image (see Figure 6). If the new image frame is a keyframe, the next step is to perform a bundle adjustment of the local map. We define the vector $\chi^{*}$ as the variable to be optimized, including the pose $T_{kw}$ of the new keyframe, the position $\rho_{i}$ of each 3D point, and the 3D position $\left\{P_{j,k},Q_{j,k}\right\}$ of each end of the line segment. Therefore, in the final step of motion estimation, we refine the camera pose and landmark position $\chi^{*}=\left\{T_{kw},\rho_{i},P_{j,k},Q_{j,k}\right\}$ by minimizing the sum of the squares of the reprojection errors:

(28) $$\chi^{*}=\underset{\chi }{\arg\min}\sum_{k\in K}\sum_{i\in P_{k}^{C}}\frac{1}{2}{∥r_{p}\left(T_{k,w},\rho_{i}\right)∥}^{2}+\sum_{k\in K}\sum_{i\in L_{k}^{C}}\frac{1}{2}{∥r_{l}\left(T_{k,w},P_{j,k},Q_{j,k},l_{j}\right)∥}^{2},$$

where *K* is the set of all keyframes in the map, $P_{k}^{C}$ is the set of landmarks associated with all the corner features, and $L_{k}^{C}$ is the set of line features observed in the kth keyframe.

In the above formula, the projection error $r_{p}$ represents the distance error between the feature position u of the image plane optimized in the previous step and the corresponding map projection point, which can be expressed as:

(29) $$r_{p}\left(T_{k,w},\rho_{i}\right)=u-\pi \left(T_{CB}T_{kw}\rho_{i}\right).$$

The processing of line segments is slightly different because we cannot simply compare the position of the endpoint because it may be shifted along the line or occluded from one frame to the next. To this end, we consider the distance between the projected endpoint of the 3D line segment and the corresponding infinite line in the image plane as an error function. In this case, the projection error $r_{l}$ of the line segment can be expressed as:

(30) $$r_{l}\left(T_{k,w},P_{j,k},Q_{j,k},l_{j}\right)=\left[\begin{array}{c} l_{j}\cdot \pi \left(T_{CB}T_{kw}P_{j,k}\right)\\ l_{j}\cdot \pi \left(T_{CB}T_{kw}Q_{j,k}\right) \end{array}\right],$$

where $P_{j,k}$ and $Q_{j,k}$ refer to the 3D endpoint of the line segment in the world coordinate system, and $l_{j}$ is the infinite line equation corresponding to the 3D line segment in the image plane, which can be obtained by the cross product between the 2D endpoints of the line segment in the image plane, i.e.,: $l_{j}=p_{j}\times q_{j}$. The reprojection error of a line segment is defined as the vector product of the projection of the two endpoints $P_{j,k}$, $Q_{j,k}$ of the 3D line segment and $l_{j}$ at the image plane.

### 3.5. Map

The task of the mapping thread is to estimate the depth information of the key frame image feature points and line segments whose depth is unknown. The depth error model of the pixel is regarded as a probability distribution, and the inverse depth of the Gauss-uniform mixture distribution (the depth value obeys the Gaussian distribution, and the probability of the outlier point obeys the Beta distribution) is called a depth filter. Each feature point serves as a seed (a pixel whose depth is not converged) and has a separate depth filter. The depth filter mainly performs the following two steps.

Initialize the seed: If a keyframe is entered, the new feature points on the keyframe are extracted, and the depth filter is initialized and placed as a seed point in a seed queue.

Update seed: If a normal frame is entered, the probability distribution of all seed points is updated with the information of the normal frame; if the depth distribution of a seed point has converged, it is placed in the map for use by the tracking thread.

For line segment features, we need to estimate the three-dimensional coordinates of the two endpoints. The LSD line segment feature is extracted in the key frame, and the endpoint is used as an initialization seed to update the seed in the non-key frame. This also incorporates line segment features into the unified framework of the depth filter.

## 4. Experimental

To evaluate the performance of the proposed algorithm, we tested our algorithm on the public visual inertial dataset EuRoC [42] and compared it with other open-source SLAM algorithms. EuRoC is stereo IMU datasets collected in the room by Swiss Federal Institute of Technology Zurich using drones. The datasets consist of two scenes, a mechanical room and a man-made common room. The hardware device includes a global shutter stereo camera with a frequency of 20 Hz and an IMU sensor with a frequency of 200 Hz. The datasets contain a total of 11 sequences, each of which provides ground-truth. In addition, the calibrated camera internal parameters and IMU-camera joint external parameters are also provided in the datasets.

The experiment was performed on a personal computer configured with an Intel Core i5-7500 CPU, 3.4 GHz × 4, 8 GB RAM. Section 4.1 compares our method with the state of art methods and gives detailed evaluation results.

### 4.1. Experimental Results

Before conducting the accuracy analysis of the experimental results, we first introduce the indicators for measuring accuracy. When evaluating the accuracy of the SLAM algorithm, there are two main indicators: Relative Pose Error (RPE) and Absolute Pose Error (APE). The relative pose error is calculated over a fixed time interval, which measures the local correctness of the estimated trajectory. Let the estimated pose be $P_{i}\in SE\left(3\right),i=1\ldots n$, the true value of the pose is $Q_{i}\in SE\left(3\right),i=1\ldots n$, and the relative pose error of the time *i* is defined as:

(31) $$E_{i}={\left(Q_{i}^{-1}Q_{i+\Delta }\right)}^{-1}\left(P_{i}^{-1}P_{i+\Delta }\right).$$

Absolute pose error: The global error of the trajectory is measured by comparing the estimated distance between the pose and the ground-truth, which can reflect the degree of deviation of the global path. Let the estimated pose be $P_{i}\in SE\left(3\right),i=1,\ldots ,n$, the true value of the pose is $Q_{i}\in SE\left(3\right),i\;=\;1,\ldots ,n$, and the absolute pose error of the time *i* is defined as:

(32) $$E_{i}=Q_{i}^{-1}P_{i}.$$

The root mean square error can be calculated using the absolute pose error at all times:

(33) $$RMSE\left(E_{i:n}\right)={\left(\frac{1}{m}\sum_{i=1}^{m}{∥trans\left(E_{i}\right)∥}^{2}\right)}^{\frac{1}{2}}.$$

We will compare the proposed method with the current state-of-the-art open-source algorithms ORB-SLAM [10] and OKVIS-mono [31]. As an extension of SVO, we also compare our algorithm with the original SVO. The experiment is unified on the data on the left image, and the estimated trajectory is saved and compared with the ground-truth. Using the open-source evaluation tool Evo [43] (github.com/MichaelGrupp/evo) to evaluate, the RPE and APE between the estimated trajectory and the ground-truth can be directly obtained, and each algorithm is evaluated 5 times on average.

**Figure 7:**
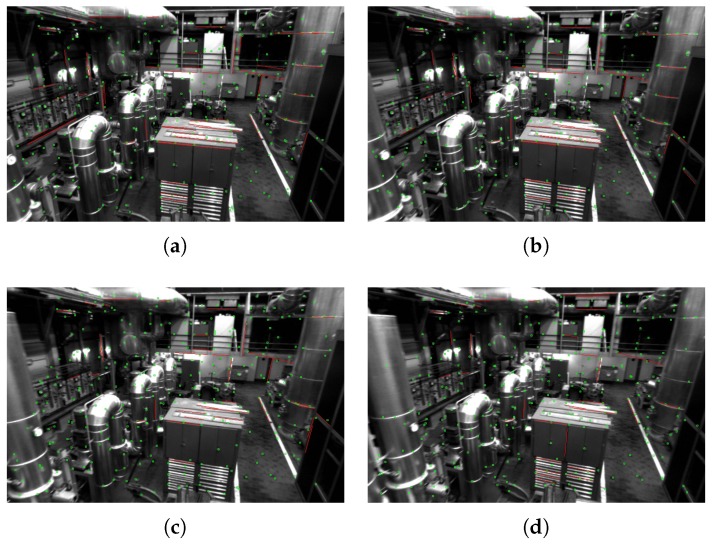
The MH_02_easy sequence tracks the point and line features of successive frames. The points are indicated in green and the segments are indicated in red. Figure (**a**–**d**) are consecutive images on the MH_02_easy sequence, drawed with point and line features which can be successfully tracked.

Next, we evaluate the tracking results of the proposed algorithm for point and line features between consecutive frames. As shown in Figure 7, the figure shows the tracking results of point and line segment features between consecutive frames on the MH_02_easy sequence. Point features are shown in green and line segments are shown in red. As can be seen from Figure 7, a large number of line features are successfully tracked between consecutive frames, which is advantageous for improving the accuracy of the pose estimation.

**Figure 8:**
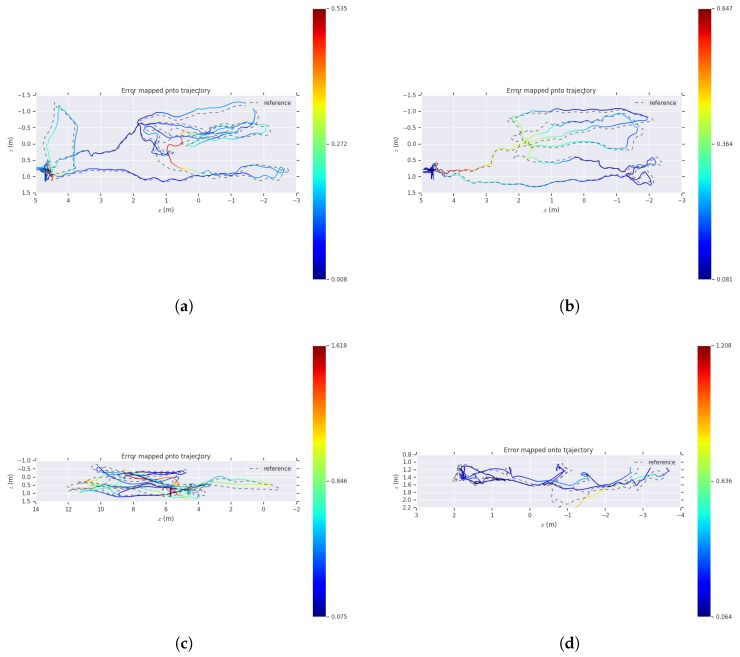
The comparison of the estimated pose trajectory and the ground-truth on several EuRoC sequences. (**a**) MH_01_easy; (**b**) MH_02_easy; (**c**) MH_03_medium; (**d**) V2_01_easy. The dashed line indicates the reference trajectory, the solid line is the trajectory estimated by our algorithm, and the color indicates the absolute pose error (APE) error from the true value.

Figure 8 shows a comparison of the estimated trajectory and the reference trajectory of our algorithm on several sequences. The dashed line indicates the ground-truth of the sequence, and the solid line is the trajectory estimated by our algorithm, and the color indicates the APE error from the true value. As we can see, our algorithm shows good performances on different sequences.

**Figure 9:**
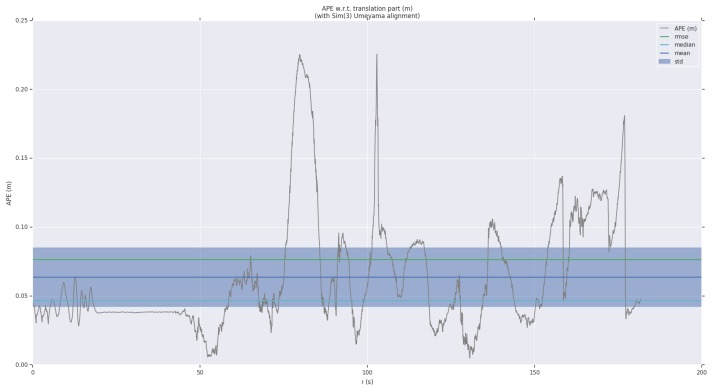
Absolute pose error of MH_01_easy sequence as a function of time.

**Figure 10:**
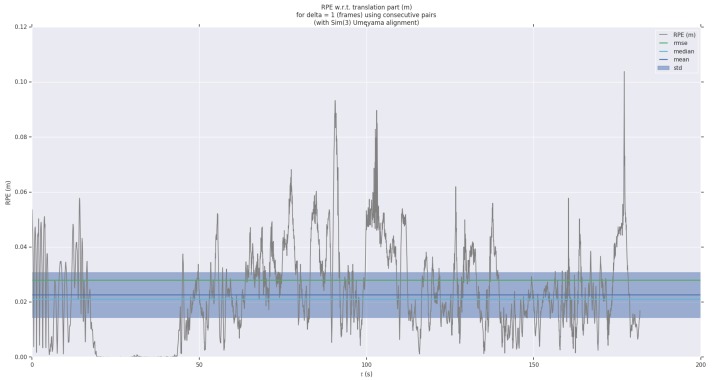
Relative pose error of MH_01_easy sequence as a function of time.

For detailed analysis, we have visualized the APE and RPE in a sequence. As shown in Figure 9 and Figure 10, we paint the APE and RPE error on MH_02_easy over time. We can analyze from this that the RPE and APE are relatively large when camera moves faster.

For quantitative evaluation, our algorithm is compared to OKVIS-mono, ORB-SLAM, and SVO. The algorithm in this paper has no loop-closure detection and global bundle adjustment optimization. For a fair comparison, compare our algorithm to ORB-SLAM and OKVIS-mono without loop-closure. As shown in Table 1, in a total of 8 sequences, our method achieved the smallest APE error in the three sequences MH_01_easy, MH_02_easy, V2_02_medium, and OKVIS-mono achieved the smallest APE error in the remaining five sequences. From the above analysis, we can achieve an absolute attitude evaluation result with OKVIS-mono, which is better than the ORB-SLAM algorithm without loop-closure. Compared to SVO, we achieved better performance in the four sequences MH_01_easy, MH_02_easy, V2_01_easy, V2_02_medium. However, SVO tracks failures in most sequences, and our algorithm can successfully track the entire trajectory, showing superior robustness.

We further estimate the results of the algorithm on the RPE and evaluate the local trajectory accuracy of the algorithm. Figure 9 shows the RPE over time in the MH_01_easy sequence. Table 2 shows the root mean error (RMSE) of the translation part of the RPE of our algorithm on 8 different sequences and compared with ORB-SLAM and OKVIS-mono. Our proposed algorithm achieves the smallest RPE error in seven sequences. It shows that the local location accuracy of the algorithm is very high.

In order to analyze the impact of loop closure on APE, we compared the ORB-SLAM algorithm with and without loop closure on APE. The results show in Table 3. It can be seen from the comparison of the presence or absence of loop-closure of the same sequence that the global error APE is reduced by adding loop-closure detection. The reason for the analysis shows that the purpose of loop-closure detection and global optimization is to reduce the cumulative error and make the system output trajectory have global consistency, which is consistent with the experimental results obtained in this paper to reduce the APE error.

### 4.2. Processing Time

Finally, we analyze the processing time comparison between the direct method of combining point and line features and the feature-based method. Table 4 shows the comparison of the mean consumption time of tracking one frame using our algorithm with other algorithms. We also recorded the runtime of each module of the algorithm in this paper. The time of each module of the algorithm in this paper is shown in Table 5.

By analyzing Table 4 and Table 5, it can be concluded that on the same hardware platform, the average time of our algorithm to process one camera frame is 16.34 ms less than the ORB-SLAM without loop-closure, which indicates that the real-time performance of the algorithm is good. This is also the advantage of the direct method compared to the feature-based method. The reason for the analysis shows that our algorithm does not need to detect and match features in every frame. Especially for line segment features, LSD line segment feature detection and LBD [44] line segment feature matching scheme is a feature time-consuming method. Our method only performs feature extraction of points and line segments on key frames in the map thread. The feature-based method ORB-SLAM requires feature matching between successive frames and between the latest frames and maps, which takes a lot of time.

### 4.3. Discussion

The algorithm in this paper can be seen as an extension of SVO, adding line segment features and motion priors. In challenging environments such as lighting changes, motion blur, and fast motion, camera tracking robustness can be improved. Compared with the feature-based method, this algorithm is based on the direct method to construct the photometric error of adjacent frames, which is free of feature extraction and matching of image frames, and can achieve fast tracking with competitive accuracy. Compared with SVO, the line segment feature can increase the number of feature tracking. When the point feature tracking is insufficient, the line segment feature can increase the number of feature tracking, increase the probability of successful consecutive frame tracking, and improve the robustness of the algorithm. Combined with the motion prior information of IMU, the accurate scale is successfully restored.

Compared with the ORB-SLAM and OKVIS-mono algorithms, the algorithm does not have loop-closure detection and global bundle adjustment module to reduce the long-term accumulated trajectory drift. Therefore, the absolute pose error (APE) of the estimated trajectory is slightly weaker, but better than the ORB-SLAM algorithm without the loop-closure detection module. However, the relative pose error (RPE) is kept at a good level, and most of the EuRoC datasets sequences are superior to ORB-SLAM and OKVIS-mono. The algorithm of this paper is accurate enough to estimate the pose change of adjacent frames, and the short-term pose estimation can achieve better accuracy. For applications that require short-term fast motion estimation, our algorithm is more suitable for such scenarios, and for map reconstruction, the ORB-SLAM algorithm is more suitable for reconstructing accurate scene maps. The algorithm in this paper is significantly faster than the other two methods under similar accuracy, so it is more suitable for lightweight applications, such as AR applications on the mobile terminal, to achieve fast tracking with a small computational load.

## 5. Conclusions

In this paper, we propose a novel visual odometry algorithm, which can be seen as an extension of SVO, combining line segment features and motion prior information. Line features help to improve system robustness in the lack of point features, such as weak textures and illumination change, so we have a more robust system. When a new camera frame is introduced, there is no need to detect and match features, achieving faster speed and less resource consumption than a feature-based approach with similar accuracy.

The direct method can quickly track line segment features and combine motion prior to obtaining accurate scales. We also provide a comparison of the proposed algorithm with the most advanced SLAM method on the EuRoC datasets, including OKVIS-mono, ORB-SLAM. The experimental results show that the proposed algorithm has a smaller RPE error, better than ORB-SLAM and OKVIS-mono, indicating better local accuracy. For the absolute pose error, the algorithm can achieve the accuracy equivalent to OKVIS-mono, which is better than the loop-closure ORB-SLAM algorithm. Compared with semi-direct method SVO, our method shows better results in half of the total sequences and has better robustness. In addition, we also evaluate the mean time that the algorithm takes to process one image frame. The results show that the algorithm has a great speed advantage and better real-time performance than ORB-SLAM and OKVIS-mono.

## Figures and Tables

**Table 1:** Evaluation results of the evo tools on different algorithms. The table shows the error in the translation part of the APE in meter. Bold numbers indicate that the estimated trajectory is closer to the true value. The results of ORB-SLAM are for reference only and do not participate in comparison. Compare our algorithm to Oriented FAST and Rotated BRIEF-Simultaneous Localization and Mapping (ORB-SLAM) (no loop closure), Open Keyframe-based Visual–Inertial SLAM (OKVIS-mono), and semi-direct VO (SVO).

	Ours	ORB-SLAM (No Loop Closure)	OKVIS-Mono	SVO
Sequences	APE (Translation)	APE (Translation)	APE (Translatin)	APE (Translation)
MH_01_easy	**0.076546**	0.61	0.394244	0.17
MH_02_easy	**0.227209**	0.72	0.309899	0.27
MH_03_medium	0.927710	1.70	**0.316398**	0.43
MH_04_difficult	1.852429	6.32	**0.432456**	1.36
MH_05_difficult	2.574021	5.66	**0.496211**	0.51
V1_01_easy	1.089041	1.35	**0.073794**	0.20
V2_01_easy	0.203892	0.53	**0.150363**	0.30
V2_02_medium	**0.192047**	0.68	0.207845	0.47

**Table 2:** Evo tool evaluation results on different algorithms. The table shows the error in the translation of the relative pose error (RPE) in meter. Bold numbers indicate that the estimated trajectory is closer to the true value.

	Ours	ORB-SLAM	OKVIS-Mono
Sequences	RPE (Translation)	RPE (Translation)	RPE (Translation)
MH_01_easy	**0.027975**	0.574031	0.068366
MH_02_easy	**0.028485**	0.467114	0.064683
MH_03_medium	**0.070834**	1.333867	0.172124
MH_04_difficult	0.173901	0.728156	**0.094539**
MH_05_difficult	**0.065546**	0.637773	0.139324
V1_01_easy	**0.027194**	0.585675	0.049214
V2_01_easy	**0.019217**	0.230216	0.044451
V2_02_medium	**0.043202**	0.625564	0.094399

**Table 3:** Evaluation results of the evo tools on ORB-SALM algorithms. The table shows the error in the translation part of the APE in meter. Bold numbers indicate that the estimated trajectory is closer to the true value.

	ORB-SLAM	ORB-SLAM (No Loop Closure)
Sequences	APE (Translation)	APE (Translation)
MH_01_easy	**0.043234**	0.61
MH_02_easy	**0.037499**	0.72
MH_03_medium	**0.036133**	1.70
MH_04_difficult	**0.062301**	6.32
MH_05_difficult	**0.065937**	5.66
V1_01_easy	**0.094841**	1.35
V2_01_easy	**0.056340**	0.53
V2_02_medium	**0.056987**	0.68

**Table 4:** The mean time to process a camera frame on a hardware platform using the Intel Core i5-7500 CPU (3.4 GHz × 4).

	Meantime (ms)
This Work	11.91
ORB-SLAM (No loop closure)	28.25
OKVIS-mono	25.16

**Table 5:** The algorithm in this pap5.

	Meantime (ms)
Pyamid Creation	0.23
Sparse Image Alignment	4.37
Feature Alignment	6.58
Pose and Structure Refinement	0.73
Total Motion Estimation	11.91
